# Adult-onset Still’s disease biological treatment strategy may depend on the phenotypic dichotomy

**DOI:** 10.1186/s13075-019-1838-6

**Published:** 2019-02-12

**Authors:** François Vercruysse, Thomas Barnetche, Estibaliz Lazaro, Emilie Shipley, François Lifermann, Alexandre Balageas, Xavier Delbrel, Bruno Fautrel, Christophe Richez, Thierry Schaeverbeke, Marie-Elise Truchetet

**Affiliations:** 1 Rheumatology Department, Centre Hospitalier Universitaire de Bordeaux, FHU ACRONIM, Service de Rhumatologie, Place Amélie Raba Léon, 33076 Bordeaux, France; 2 Centre Hospitalier Universitaire de Bordeaux, FHU ACRONIM, Service de Médecine Interne, Avenue Magellan, 33600 Pessac, France; 3 Centre Hospitalier de Dax, Service de Rhumatologie, Boulevard Yves du Manoir, 40100 Dax, France; 4 Centre Hospitalier de Dax, Service de Médecine Interne, Boulevard Yves du Manoir, 40100 Dax, France; 5 Centre Hospitalier de Pau, Service de Rhumatologie, 4 Boulevard Hauterive, 64000 Pau, France; 6 Centre Hospitalier de Pau, Service de Médecine Interne, 4 Boulevard Hauterive, 64000 Pau, France; 7 Centre Hospitalier Universitaire de Paris, Hôpital Pitié Salpêtrière, Service de Rhumatologie, Boulevard de l’Hopital, 75013 Paris, France

**Keywords:** Adult-onset Still’s disease, Tocilizumab, Anakinra, Treatment strategy, Biologics

## Abstract

**Objectives:**

Adult-onset Still’s disease (AOSD) phenotype appears to be dichotomized in systemic or chronic articular forms. As biologicals and particularly interleukin (IL)-1 and IL-6 blockers play a more and more prominent role in the treatment, their place requires clarification. This study aimed to identify factors predictive of treatment response to anakinra or tocilizumab and investigate whether the choice of biotherapy and delays in the initiation of biotherapy influenced the likelihood of steroid discontinuation.

**Methods:**

A multicenter exploratory retrospective study included all patients diagnosed with AOSD and receiving biological treatments in three regional hospitals until 2018. Clinical and biological characteristics at diagnosis and treatment-related data were collected. The nonparametric Mann-Whitney test was used to perform univariate analysis for quantitative variables, and Fisher’s exact test was used for qualitative variables.

**Results:**

Twenty-seven patients were included. All but one patient achieved remission with either anakinra or tocilizumab. Treatment responses depended on disease phenotype: the presence of arthritis and a chronic articular phenotype were associated with a substantial response to tocilizumab with *p* = 0.0009 (OR 36 [2.6–1703]) and *p* = 0.017 (OR 10 [1.22–92.6]), respectively, whereas the systemic form and the absence of arthritis were associated with a substantial response to anakinra with *p* = 0.0009 (OR 36 [2.6–1703]) and *p* = 0.017 (OR 10 [1.22–92.6]), respectively. Tocilizumab increased the likelihood of corticosteroid withdrawal (*p* = 0.029) regardless of delays in initiation or when it was initiated relative to other treatment in the overall therapeutic strategy.

**Conclusion:**

This study highlights the therapeutic implications of the phenotypic dichotomy of AOSD and should help us better codify AOSD treatment.

## Key messages

- What is already known about this subject?
  - o The phenotype of adult-onset Still’s disease appears to be dichotomized into systemic or chronic articular forms.
  - o Biological treatments are dominated by IL-1 or IL-6 receptor inhibitors.
- What are the new lines of research suggested by this study?
  - o The presence of arthritis and a chronic articular phenotype may be associated with a preferential response to tocilizumab, whereas the systemic form may be associated with a preferential response to anakinra.
  - o Tocilizumab increases the likelihood of corticosteroid withdrawal regardless of delays in its initiation or when it is initiated relative to other treatment in the overall therapeutic strategy
- How might this impact clinical practice?
  - o These results should help us better codify the treatment strategy for adult-onset Still’s disease.

## Introduction

Adult-onset Still’s disease (AOSD) is a rare systemic inflammatory disorder. Its annual incidence is estimated to be between 0.16 and 0.62 per 100,000 persons globally [1], and its prevalence approximately 1–24 per million [2].

AOSD is an autoinflammatory disease resulting from multifactorial causes such as genetic factors and immunological imbalances involving macrophage, neutrophil, and inflammasome hyperactivation or dysfunction [3].

Recent studies suggest that there is a dichotomy between two major phenotypes of AOSD: a systemic pattern that presents with an acute onset associating high fever, skin rash, hematopoietic signs, and sometimes visceral damage; and a chronic articular profile in which polyarthritis prevails [4, 5]. This phenotypic dichotomy is highlighted by recent studies showing that there are different cytokine imbalances in these two phenotypes. Although results may vary, this imbalance would sometimes favor interleukin (IL)-1β, IL-18, and interferon (IFN)-γ production in the systemic form, whereas in the chronic articular form, IL-6, IL-8, tumor necrosis factor (TNF)-α, and IL-17 production would predominate [6–12].

The prognosis of AOSD is unpredictable as it can manifest with polyarticular erosive disease or life-threatening manifestations such as reactive hemophagocytic lymphohistiocytosis (rHLH). The disease course is also very polymorphic, ranging from a monophasic flare to polycyclic or chronic forms. This clinical and prognostic polymorphism presents a major challenge and makes it difficult to predict the outcome at the time of diagnosis and to determine appropriate management.

Therapy is therefore usually empirical, and beside methotrexate, biologics have played an increasing role in the chronic or refractory forms. A recent meta-analysis suggested that IL-1 and IL-6 receptor inhibitors are the most effective biotherapies for AOSD [13] and have a strong steroid-sparing effect. The systemic phenotype has been suggested to respond preferentially to IL-1 receptor inhibitors, such as anakinra, while the chronic articular form shows better responses to IL-6 receptor inhibitors, such as tocilizumab [6, 7, 14–17].

This exploratory study therefore aimed to identify initial clinical and/or biological factors that were predictive of a preferential response to anakinra or tocilizumab. Secondary objectives were to investigate whether the choice of biotherapy (anakinra or tocilizumab) and their delay in initiation influenced the likelihood of steroid discontinuation and to describe the clinical and biological phenotypes of patients with AOSD.

## Methods

### Study design and data collection

A multicenter exploratory retrospective study of all patients diagnosed with AOSD at Bordeaux teaching hospital and Pau and Dax peripheral hospitals until 2018 was conducted. Patients included had to be 16 years old or older, to be diagnosed with AOSD, to meet the Yamaguchi and/or Fautrel criteria, to have a chronic or refractory disease course, and to receive or have received a biological disease-modifying antirheumatic drugs (bDMARD). Exclusion criteria were age younger than 16 years old, a diagnosis of systemic-onset juvenile idiopathic arthritis (SoJIA), an exclusion of AOSD diagnosis during disease course, Yamaguchi and Fautrel criteria not met, lack of data, and absence of bDMARD during disease course.

Patients with AOSD were identified via ICD-10 code, via a diagnostic grid that automatically references autoimmune and inflammatory diseases at Bordeaux University Hospital, via the bioinformatics services of each hospital, and via direct contact with the specialists treating the patients. Data regarding clinical and biological characteristics at diagnosis and treatment details were collected using a standard data extraction form. All the information was double-checked for any discrepancies.

The following demographic and clinical AOSD-related characteristics were collected: sex, date of birth, age at diagnosis, year of diagnosis, duration of disease, duration of disease at first bDMARD initiation, smoking habits, personal or familial history of rheumatic autoimmune inflammatory or neoplastic disease, type of Still’s disease-related rash if present, arthralgia, arthritis, hands affected or not, early morning stiffness, myalgia, fever characteristics, general deterioration and weight loss > 4 kg, lymphadenopathy, pharyngitis, angina, and visceral involvement (pleuritis, pericarditis, pulmonary hypertension).

The following biological characteristics were recorded: leukocytosis > 10,000/mm^3^, neutrophilia > 8000/mm^3^, eosinophilia > 500/mm^3^, anemia with hemoglobin < 12 g/dl, thrombocytosis > 400,000/mm^3^, increased C-reactive protein (CRP) levels, maximum CRP at diagnosis, decreased albumin, increased liver enzymes, increased creatine kinase, increased lactate dehydrogenase (LDH), increased ferritin, decreased glycosylated ferritin, autoimmunity markers (antinuclear antibodies (ANA), antideoxyribonucleic acid, anti-extractable nuclear antigen, rheumatoid factor, antiβ2GP1, lupus anticoagulant, anticardiolipin), disseminated intravascular coagulation markers, and rHLH markers (cytopenia, hypertriglyceridemia, hypofibrinogenemia, hemophagocytosis in the bone marrow).

Patients were classified as having two distinct disease patterns: the systemic form or the chronic articular form. In the absence of consensual criteria in this regard, this classification was based on expert opinions that was often spelled out in the medical reports and was confronted to disease course outcome, which was available due to the retrospective nature of this study.

### Outcome measurements

The effectiveness of treatment was defined as follows: effective treatment was considered when all initial symptoms and biological anomalies had resolved, partially effective treatment was considered when all but one initial symptom or biological anomaly had resolved, and ineffective treatment was considered when two or more initial symptoms or biological anomaly persisted. The attending doctors assessed disease flares and relapses.

Treatment lines were assessed as follows: nonsteroidal anti-inflammatories (NSAIDs) were not considered a treatment modality for chronic and refractory AOSD, but corticosteroids, conventional synthetic disease-modifying antirheumatic drugs (csDMARD) and especially methotrexate (both galenic forms taken together) and cyclosporine, and bDMARD (anakinra, tocilizumab, canakinumab, infliximab, and etanercept) were considered treatment lines.

The outcome of interest for the univariate analysis of predictive factors was the full effectiveness of the treatment as defined above, including both systemic and articular manifestations.

### Statistical analysis

Quantitative variables are presented as mean with standard deviation or as median with interquartile range, and qualitative variables are presented as percentages. Analysis was performed on the following variables: all the clinical and biological manifestations included in the Yamaguchi or Fautrel criteria, common demographic characteristics, and all the symptoms or biological abnormalities usually observed during AOSD according to the literature and our personal experience. The nonparametric Mann-Whitney test was used to perform a univariate analysis of quantitative variables, and the Fisher exact test was used for qualitative variables. Calculations were performed with STATA 13.1 SE software. *P* values less than 0.05 were considered significant.

## Results

### Patients’ characteristics

Twenty-seven patients were included in this study: 21 from Bordeaux teaching hospital, 4 from Dax hospital, and 2 from Pau hospital. Patient characteristics at diagnosis are described in Table 1.

**Table 1:**
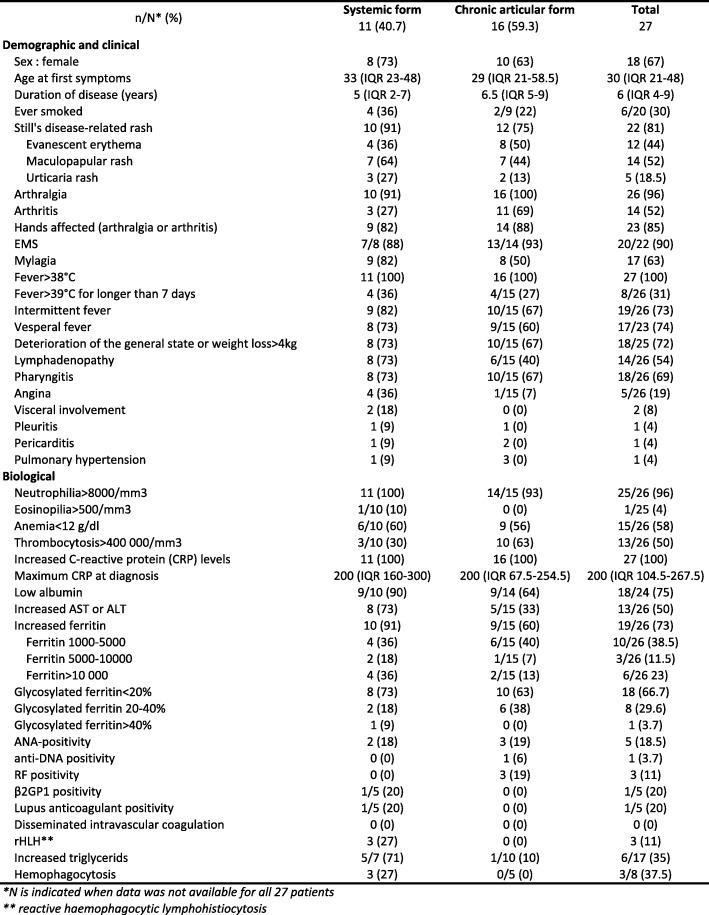
Characteristics of the patients included in the study

All patients fulfilled both the Yamaguchi and Fautrel criteria. No patients had any personal or familial history of rheumatic, autoimmune, inflammatory, or neoplastic disease. All patients presented with a fever above 38 °C, mainly intermittent and vesperal fevers. The next most prevalent symptoms were arthralgia (96%), arthritis (52%), skin rash (81%), general deterioration (72%), and ear-nose-throat (ENT) symptoms (69%).

Eleven (40.7%) patients were classified as systemic form of disease, and 16 (59.3%) as chronic articular form.

All patients presented with elevated CRP levels. The next most commonly observed anomalies were neutrophilia (96%), low albumin (75%), and increased ferritin (73%). No patients presented with increased creatine kinase (CK), positive extractable nuclear antigen (ENA), positive antineutrophil cytoplasmic antibody (ANCA), positive anticitrullinated protein antibody (ACPA), or positive anticardiolipin. Nine patients were either ANA-positive (five, 1/160), anti-DNA positive (one), or rheumatoid factor (RF)-positive (three) at diagnosis, but these results were judged clinically insignificant because lower than two times the upper normal range and never associated with symptoms concordant with SLE, APLS, or RA and were all controlled to be negative. Furthermore, all patients included in the study met both Yamagushi and Fautrel criteria.

The median duration of disease was 5 years (IQR 2–7) and 6.5 years (IQR 5–9) for the systemic and chronic articular forms, respectively.

All patients presented a chronic refractory form of the disease, which required the off-label use of bDMARD because of failures of corticosteroids and csDMARD or necessity to reduce or stop corticosteroids.

### Biological treatment efficacy

Table 2 presents the detailed responses to anakinra and tocilizumab among the patients included in the study. Twenty-six of 27 patients (96.3%) achieved remission with either anakinra or tocilizumab. The median delay in the introduction of the first biotherapy was 5 months (interquartile range (IQR), 1–21 months): this delay was median 1 month (IQR, 0–6) in the systemic form and median 17 months (IQR, 4–38) in the chronic articular form.

**Table 2:**
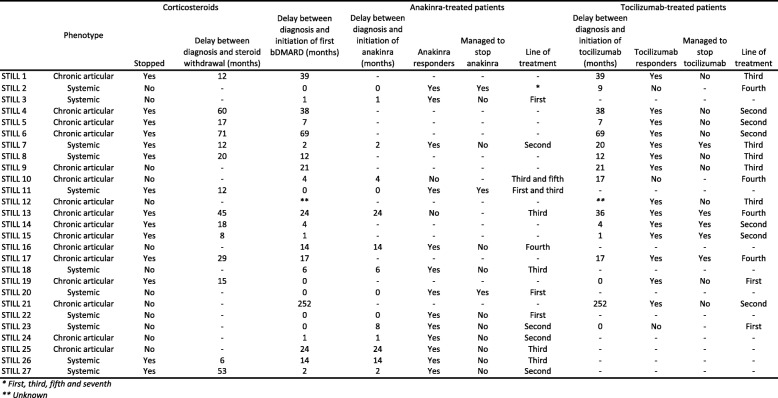
Delay in corticosteroids withdrawal and detailed responses to anakinra and tocilizumab among the patients included in the study

A total of 15 patients received anakinra; 5 as a first-line treatment, 4 as a second-line treatment, 5 as a third-line treatment, and 1 as a fourth-line treatment. The median delay between diagnosis and the introduction of anakinra was 1.5 months (IQR, 0–14). Thirteen (86.7%) patients were responders. The 2 anakinra nonresponders received anakinra as a third-line treatment: one had previously failed to respond to tocilizumab, and the other later responded to tocilizumab and even managed to stop it.

A total of 17 patients received tocilizumab, 2 as a first-line treatment, 6 as a second-line treatment, 5 as a third-line treatment, and 4 as a fourth-line treatment. The median delay between diagnosis and the introduction of tocilizumab was 17 months (IQR, 4–39 months). Fourteen patients (82.4%) were responders. Among the 3 tocilizumab nonresponders, one presented with a systemic form and received tocilizumab as a first-line treatment but later responded to anakinra, one presented with a systemic form and received tocilizumab as a fourth-line treatment after responding to anakinra but was switched because of a suspicion of anakinra-induced toxidermia, and one presented with a chronic articular form and received tocilizumab as a fourth-line treatment after failure of anakinra.

The difference of median duration of disease at the introduction of the first bDMARD in the anakinra-treated patients group and in the tocilizumab-treated patients group was statistically significant (*p* = 0.4457): median 2 months (IQR, 0.25–12 months) in the anakinra-treated patients and median 14.5 (IQR, 3.25–38.25 months) in the tocilizumab-treated patients.

Eight patients (29.6%) managed to stop their biological treatment without relapse at the last known visit which ranged from 6 to more than 24 months after biological treatment discontinuation: 3 of 13 (23%) anakinra responders and 5 of 14 (35.7%) tocilizumab responders. The difference in delays in initiation of anakinra or tocilizumab was not statistically significant between those who managed to stop their biotherapy without relapse versus those who could not stop their biotherapy or those who relapsed, with a median delay of 3 months (IQR, 1.25–13.75) versus 8.5 months (IQR, 1–23.25), respectively (*p* = 0.61).

Five patients received both biotherapies. Two of these patients presented with a systemic form and were tocilizumab nonresponders but anakinra responders, one presented with a chronic articular form and was anakinra nonresponder but tocilizumab responder, one presented with a chronic articular form and did not respond to either biotherapy, and one patient presented with a systemic form and responded to both biotherapies; this patient first responded to anakinra but corticosteroids could not be tapered so he was switched to tocilizumab in order to successfully stop corticosteroids.

One patient received canakinumab and failed to respond but was anakinra responder. Three patients received antitumor necrosis factor (TNF) agents but these treatments were not efficacious (2 etanercept, 1 infliximab): two of them presented a chronic articular form and one a systemic form.

One of 27 (3.7%) patients did not respond to either biotherapy (anakinra then tocilizumab) and died of a probably infectious-induced AOSD flare.

### Predictive factors for preferential responses to anakinra or tocilizumab

Patients who responded to either biotherapies or no biotherapy were excluded from this analysis (*n* = 2). Table 3 describes the univariate analysis for potential predictive factors of a good therapeutic response to anakinra or tocilizumab. The outcome of interest was the full effectiveness of the treatment, defined as the resolution of all initial systemic and articular symptoms and biological anomalies.

**Table 3:**
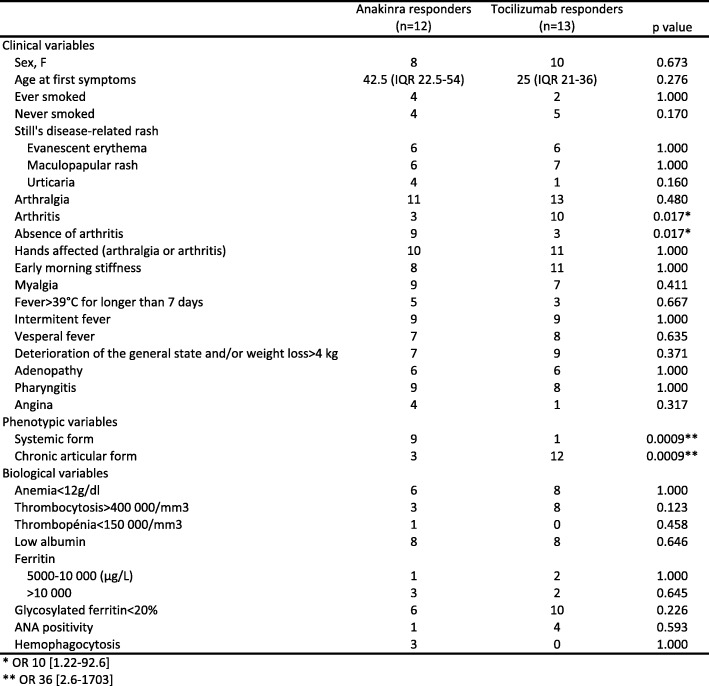
Univariate analysis of predictive factors for a good therapeutic response to anakinra or tocilizumab (*n* = 25)

A chronic articular phenotype and the presence of arthritis were associated with a substantial response to tocilizumab: 12 of the 14 patients presenting a chronic articular phenotype responded to tocilizumab whereas 3 of the 13 patients presenting a systemic phenotype responded to tocilizumab (*p* = 0.0009, OR 36 [2.6–1703]) and 10 of the 14 patients presenting arthritis responded to tocilizumab whereas 3 of the 13 patients without arthritis responded to tocilizumab (*p* = 0.017, OR 10 [1.22–92.6]).

A systemic form and the absence of arthritis were associated with a substantial response to anakinra: 1 of the 14 patients presenting a chronic articular phenotype responded to anakinra whereas 9 of the 13 patients presenting a systemic phenotype responded to anakinra (*p* = 0.0009, OR 36 [2.6–1703]) and 3 of the 14 patients presenting arthritis responded to anakinra whereas 9 of the 13 patients without arthritis responded to anakinra (*p* = 0.017, OR 10 [1.22–92.6]).

No difference in biological variables was statistically predictive of treatment response, although the presence of thrombocytosis seemed to be preferentially associated with a better response to tocilizumab than to anakinra (*n* = 8/14 vs. *n* = 3/13, *p* = 0.123).

### Likelihood of steroid withdrawal depending on the specific biotherapy and delays in initiation of biotherapy

All patients received corticosteroids at diagnosis. Among the 26 patients in remission, 14 (53.8%) achieved corticosteroid withdrawal within a mean delay of 27 months, 10 (71.4%) were on tocilizumab, and 4 (28.6%) were on anakinra (Table 2).

Table 4 presents the likelihood of corticosteroid withdrawal depending on treatment with anakinra and tocilizumab: tocilizumab was associated with an increased likelihood of corticosteroid withdrawal (*p* = 0.029), whereas anakinra did not seem to increase the likelihood of corticosteroid withdrawal (*p* = 1.000).

**Table 4:**
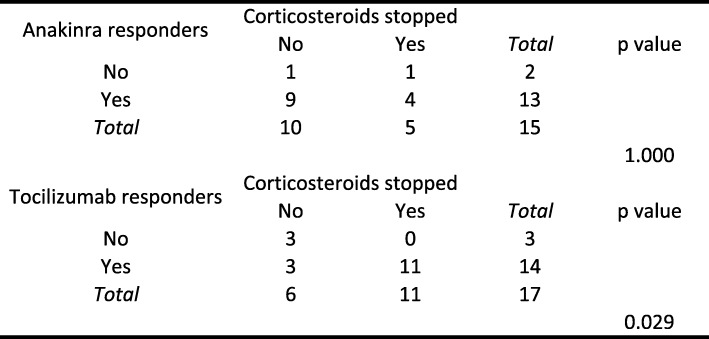
Likelihood of corticosteroid withdrawal stratified by biotherapy

A shorter delay before the initiation of anakinra or tocilizumab was not associated with a greater likelihood of corticosteroid withdrawal among biotherapy responders. Among anakinra responders, the difference in the median delay from diagnosis to anakinra initiation between those who achieved steroid withdrawal (*n* = 4) and those who did not (*n* = 10) was not statistically significant (*p* = 0.5767): the median delay was 2.5 months (IQR, 0–8 months) among those who did not stop corticosteroids versus 2 months (IQR, 2–14 months) among those who stopped corticosteroids. Among tocilizumab responders, the difference in the median delay from diagnosis to tocilizumab initiation between those who achieved steroid withdrawal (*n* = 11) and those who did not (*n* = 5) was not statistically significant (*p* = 0.9548): the median delay was 17 months (IQR, 9–21 months) among the tocilizumab responders who did not stop corticosteroids versus 17 months (IQR, 4–38 months) among those who stopped corticosteroids. Initiating tocilizumab as the first-line treatment rather than as a second-line or later treatment did not improve the likelihood of corticosteroid withdrawal (*n* = 1/2 vs. *n*= 11/15, *p* = 1.000). In addition, initiating tocilizumab as a first- or second-line treatment rather than as a third-line or later treatment also did not improve the likelihood of steroid withdrawal (*n* = 6/8 vs. *n* = 5/9, *p* = 0.619); similarly, initiating tocilizumab as a first-, second- or third-line treatment did not improve the likelihood of steroid withdrawal compared to that from tocilizumab used as a fourth-line treatment (*n* = 9/13 vs. *n* = 2/4, *p* = 0.584).

## Discussion

This retrospective study was conducted to evaluate bDMARD strategies in the management of AOSD, with a special attention to predictive factors of their efficacy and their effect on corticosteroid sparing. All but one patient achieved remission with either anakinra or tocilizumab. Our results support the dichotomy of AOSD in two phenotypes: treatment responses to bDMARD seemed to depend on disease phenotype, the presence of arthritis, and a chronic articular phenotype were associated with a substantial response to tocilizumab, whereas the systemic form was associated with a substantial response to anakinra. In our cohort, anakinra did not increase the likelihood of corticosteroid withdrawal but tocilizumab did, regardless of delays in initiation, or when it was initiated relative to other treatment in the overall therapeutic strategy.

The clinical and biological characteristics of the patients included in this study seem similar to those of AOSD patients reported in the literature [18], with the exception of life-threatening conditions, which have been estimated to affect as many as 15–20% of AOSD patients, while only 8% were affected in this study.

Multivariate analysis was not performed because of the sample size, a limitation inherent to rare diseases such as AOSD. As a consequence, we lacked patients who received both biotherapies in each phenotype. But rather than aiming to look for significant differences and to draw definite conclusions, the goal of this study was to establish a reference for future large-scale studies and compile data for registries to confirm the results.

The distinction between systemic and chronic articular forms is not always straightforward, and no consensual criteria exist to distinguish these two phenotypes. For example, a systemic form progressing for several years, who received corticosteroids for a long time and who benefited from bDMARD late in disease course, might be confused for a chronic articular form.

These results highlight the excellent efficacy of anakinra and tocilizumab in AOSD, regardless of the phenotype, but show some differences between them. However, the retrospective nature of the study is a major limitation to compare these two drugs. The choice of biotherapy may have been different depending on their availability on the year of diagnosis: anakinra was launched on the market several years before tocilizumab and the effects of anakinra and tocilizumab on AOSD were not simultaneously described.

One could argue that clinicians may have influenced the choice of biotherapy by preferentially prescribing tocilizumab to patients presenting with arthritis or a chronic articular form, analogous to rheumatoid arthritis, and therefore distorted the results. Comparing these two bDMARDs is impossible outside a randomized controlled trial (RCT), and none ever demonstrated that tocilizumab should be preferentially prescribed to AOSD patients with arthritis [5, 19, 20].

Yet the following results reinforce our findings. All but three of the patients included in this study were diagnosed in 2009 or later, and these three received anakinra or tocilizumab after both their efficacies were described in AOSD.

A major result of our study is that anakinra responders more commonly did not have arthritis, as the absence of arthritis was statistically associated with a better response to anakinra (*p* = 0.017). Tocilizumab also showed less efficiency on the systemic form. Although our results cannot imply definitive conclusions on the fact that one biotherapy is more effective than the other in each phenotypes because of the lack of patients who received both biotherapy in each phenotype, this result supports our clinical feeling and the notion that even if clinicians’ choices may have been influenced by analogy to rheumatoid arthritis, their insights seem to be founded.

While there is no doubt about anakinra’s dramatic efficacy on AOSD in general and particularly on the systemic phenotype [2, 18, 19, 21–27], whether anakinra is effective for joint involvement is a subject of debate as data seem to be inconsistent [4, 21–24, 28, 29]. Some studies reported a substantial efficacy [5, 23, 24], when others suggest otherwise [21, 28, 30]. The largest retrospective study investigating anakinra in AOSD showed a significant improvement on articular manifestations (DAS28 falling from 4.7 ± 1.2 to 1.7 ± 0.9 after 12 months) but 14.4% of the patients still presented arthritis after 12 months of treatment [24]. Notably, they found no difference in delay of response to therapy between the two phenotypes and did not observe any difference in the type of response in the two patterns of the disease. In our study, 5 patients presenting with a chronic articular form received anakinra: 3 responded to anakinra and never received tocilizumab, one was an anakinra nonresponder but responded to tocilizumab, and one responded to anakinra but had to be switched to tocilizumab in order to stop the corticosteroid. Definitive conclusions about anakinra’s efficacy on AOSD’s joint involvement therefore seem still premature.

The absence of demonstrated steroid-sparing with anakinra in this study is questionable as it demonstrated a steroid-sparing effect in several other studies [22, 23, 25]. First, the evaluation in our study was based on the rate of steroid withdrawal, not on a steroid-sparing effect as prednisone dosage was not available. Secondly, a channeling bias exists: the acute onset of the systemic phenotype and its potential life-threatening complications might cause the clinicians to be more cautious while tapering corticosteroids, and the tocilizumab-induced fall in CRP might reassure them. Furthermore, anakinra was made available before tocilizumab: the progressively known dramatic efficacy of anakinra may have reassured the clinicians and entice them into a faster corticosteroids tapering when tocilizumab was made available. In addition, three patients presenting a systemic form in our study were able to taper corticosteroids down to 5 mg daily but the clinicians opted to maintain this dosage as a long-term therapy. Both the management and the place of corticosteroids in AOSD treatment strategy should maybe differ based on the differences between the two phenotypes.

No patients in this study responded to anti-TNF blockers, whatever the phenotype. The efficacy of these medications in AOSD is controversial, but some studies suggest that they have a place in the treatment of the chronic articular form [31–33], while others have showed rather disappointing results [19, 20, 34]. The excellent efficacy and clear steroid-sparing effect of tocilizumab determined in our study, concurring with the findings of a recent meta-analysis showing that AOSD patients treated with tocilizumab had a pooled remission rate of approximately 85% regardless of the phenotype [35], and of the first RCT investigating tocilizumab in AOSD [36], should make clinicians prioritize tocilizumab to anti-TNF blockers for patients with a chronic articular form of AOSD. Canakinumab showed some inconsistent results in AOSD [37–39] but is currently being investigated for its efficacy on AOSD’s joint involvement (NCT02204293). No patient in this study received abatacept or rituximab; the data are very limited but some authors reported efficacy in patients who fail to respond to TNF, IL-1, or IL-6 inhibition [40–45].

Physiopathological data may highlight the efficacy of IL-17 or IL-18 blockade in AOSD. A recent study showed that IL-18 inhibition using the recombinant human IL-18-binding protein tadekinig alpha is a therapeutic option in patients with AOSD in a phase 2 open-label trial [46]. IL-17 [47] and INF-γ [48] blockade have not been studied yet but should be in the near future. Janus kinase (JAK) inhibitors may also be of interest, particularly in the chronic articular form of AOSD.

The two main goals when initiating a bDMARD in AOSD should be a complete remission and corticosteroids withdrawal or significant tapering.

This study does not reveal definitive answers regarding whether an early introduction of biologics improves the general prognosis of AOSD. We believe that the early use of biologics in AOSD patients is worth considering. It is our impression that the earlier biologics are used, the shorter and smoother the disease course is. Similarly, other authors have suggested that biotherapies should be initiated earlier in the course of AOSD [13, 18], arguing that earlier initiation may improve the prognosis mainly via a steroid-sparing effect. Notably, the results of this study suggest that the steroid-sparing effect of biologics is not influenced by delays in initiation of biological therapy.

One-quarter to one-third (29.6%) of our patients managed to stop biologics despite chronic or refractory forms of AOSD, raising the question of how and when to stop biologics in these patients. A channeling bias exists, as no predefined corticosteroids tapering strategy could be set due to the retrospective nature of this study but patients who managed to stop anakinra had all achieved remission for at least 6 to 12 months, had initially stopped corticosteroids, and then all underwent progressive reduction in their biological treatments before definitive discontinuation. Among patients who managed to stop tocilizumab, corticosteroids had always previously been stopped, and tocilizumab dosage was progressively decreased when it had been administered by perfusion or tapered when it had been administered by injections. The high percentage of patients who successfully discontinued biological treatments in our study should encourage clinicians to consider tapering biologics among patients without visceral involvement who are in clinical and biological remission for 6 to 12 months.

The first evidence-based clinical practice guidelines for the management of AOSD [49] emphasized the use of IL-6 inhibitors in the treatment of AOSD, but IL-1 inhibitors were judged to be “weakly recommended as a therapeutic agent against refractory AOSD” although one RCT and several case reports suggest otherwise. A proposal may be to treat a first flare of AOSD with corticosteroids and to introduce biologics in cases of steroid-refractory flares or as early as the second flare of AOSD. In that case, anakinra should be preferred when faced with the systemic form of AOSD and tocilizumab in patients with the articular form. The well-known idea of a “window of opportunity” in rheumatoid arthritis may also be valid in AOSD, with the objective being to prevent the development of a self-perpetuating “cytokine storm.”

We need large-scale studies, RCTs, and international collaborative registries regarding this multifaceted disease. As emphasized in a recent editorial, these studies will have to be more precise about evaluating the patients’ disease course and particularly their phenotypes in order to concentrate research on the subgroup of patients with a chronic or refractory form of the disease [50]. A national French cohort, the French Adult and Childhood Onset Still Disease Cohort (RaDiCo-ACOSTILL), was recently opened for inclusion and will surely help to shed light on these issues in the near future.

## Conclusion

This study supports the dichotomy of AOSD in two phenotypes and introduces the idea that treatment response to bDMARD may depend on disease phenotype. The presence of arthritis and a chronic articular phenotype seem to be associated with a substantial response to tocilizumab, whereas the systemic form seems to be associated with a substantial response to anakinra. Although further research is needed, this exploratory study highlights the therapeutic implications of the phenotypic dichotomy of AOSD. These results should help us better codify treatment strategies for AOSD.

## Abbreviations

ANA: Antinuclear antibodies
ANCA: Antineutrophil cytoplasmic antibody ACPA Positive anticitrullinated protein antibody
Anti-DNA: Antideoxyribonucleic acid
Anti-ENA: Anti-extractable nuclear antigen
AOSD: Adult-onset Still’s disease
APLS: Anti-phospholipid syndrome
bDMARD: Biological disease-modifying antirheumatic drugs
CK: Creatine kinase
CRP: C-reactive protein
csDMARD: Conventional synthetic disease-modifying antirheumatic drugs
ENT: Ear-nose-throat
IL: Interleukin
INF: Interferon
IQR: Interquartile range
JAK: Janus kinase
LDH: Lactate dehydrogenase
NSAIDs: Nonsteroidal anti-inflammatories
RCT: Randomized controlled trial
RF: Rheumatoid factor
rHLH: Reactive hemophagocytic lymphohistiocytosis
SLE: Systemic lupus erythematosus
SoJIA: Systemic-onset juvenile idiopathic arthritis
TNF: Tumor necrosis factor
